# Supplementary material for: Construction and investigation of multi-enzyme immobilized matrix for the production of HFCS
Source: PLoS One. 2024 Feb 16;19(2):e0292931. doi: 10.1371/journal.pone.0292931 (PMC10871492; doi:10.1371/journal.pone.0292931)
Supplement: Attachment — Submitted filename: Response to Reviewers.docx [file pone.0292931.s002.docx]

Re: PLOS ONE Decision: Revision required [PONE-D-23-22990] - [EMID:9cad34958b1a9769]

Title: " Construction and Investigation of Multi-enzyme Immobilized Matrix for the Production of HFCS "

 Author(s): Sabbir Janee^1^, Shatabdy Saha^1^, Sabrina Sharmin^2^, A.Q. Fuad Hasan^1^, Umme Salma Zohora^1^*,* Ripa Moni^1^, Md. Zahidul Islam^1,#^ and Mohammad Shahedur Rahman^1,3^

September 10, 2023

To,

Saleh Ahmed Mohamed
Academic Editor
PLOS ONE

Dear Sir,

Thank you very much for your letter on our manuscript (PONE-D-23-22990) dated on 21 July 2023. In accordance with the reviewers’ comments, we made the revised version of the manuscript. In the manuscript with highlights, the revised portions according to the reviewers are indicated by red letters. We have provided the minimal underlying data as an Excel file from which we have derived our interpretation of the study. Would you please take care of the revised manuscript?

Thank you very much.

Sincerely yours,

Dr. Md. Zahidul Islam

Associate Professor

Department of Biotechnology and Genetic Engineering,

Jahangirnagar University, Savar, Dhaka-1342, Bangladesh.

Phone: +88-01787-086965

E-mail: zahidul@juniv.edu

**Answer to the Journal requirements**

First of all, we wish to appreciate for your valuable comments and suggestions. We revised our manuscript according to the comments and answers to the given questions are as follows.

*1. Please ensure that your manuscript meets PLOS ONE's style requirements, including those for file naming.*

Thank you very much for your comment. According to the comment, we have revised our manuscript in pursuance of the PLOS ONE style templates.

2. Thank you for stating the following financial disclosure:

“The funders had no role in study design, data collection and analysis, decision to publish, or preparation of the manuscript”

At this time, please address the following queries:

a) Please clarify the sources of funding (financial or material support) for your study. List the grants or organizations that supported your study, including funding received from your institution.

In this research project, we did not receive any funds or grants.

*b) State what role the funders took in the study. If the funders had no role in your study, please state: “The funders had no role in study design, data collection and analysis, decision to publish, or preparation of the manuscript.”*

We did not receive any funds from any funder. Therefore, the funders had no role in study design, data collection and analysis, decision to publish, or preparation of the manuscript.

*c) If any authors received a salary from any of your funders, please state which authors and which funders.*

Not Applicable

*d) If you did not receive any funding for this study, please state: “The authors received no specific funding for this work.”*

The authors received no specific funding for this work.

3. "In your Data Availability statement, you have not specified where the minimal data set underlying the results described in your manuscript can be found. PLOS defines a study's minimal data set as the underlying data used to reach the conclusions drawn in the manuscript and any additional data required to replicate the reported study findings in their entirety. All PLOS journals require that the minimal data set be made fully available. For more information about our data policy, please see http://journals.plos.org/plosone/s/data-availability.

"Upon re-submitting your revised manuscript, please upload your study’s minimal underlying data set as either Supporting Information files or to a stable, public repository and include the relevant URLs, DOIs, or accession numbers within your revised cover letter. For a list of acceptable repositories, please see http://journals.plos.org/plosone/s/data-availability#loc-recommended-repositories. Any potentially identifying patient information must be fully anonymized.

Important: If there are ethical or legal restrictions to sharing your data publicly, please explain these restrictions in detail. Please see our guidelines for more information on what we consider unacceptable restrictions to publicly sharing data: http://journals.plos.org/plosone/s/data-availability#loc-unacceptable-data-access-restrictions. Note that it is not acceptable for the authors to be the sole named individuals responsible for ensuring data access.

We will update your Data Availability statement to reflect the information you provide in your cover letter.

We have provided the minimal underlying data as an Excel file from which we have derived our interpretation of the study.

*4. Please review your reference list to ensure that it is complete and correct. If you have cited papers that have been retracted, please include the rationale for doing so in the manuscript text, or remove these references and replace them with relevant current references. Any changes to the reference list should be mentioned in the rebuttal letter that accompanies your revised manuscript. If you need to cite a retracted article, indicate the article’s retracted status in the References list and also include a citation and full reference for the retraction notice.*

According to the comment, we have updated the references using EndNote software.

**Answer to the Reviewer-1 comments:**

First of all, we wish to appreciate for your valuable comments and suggestions. Here is our reply to your comments.

*1. Is the manuscript technically sound, and do the data support the conclusions?

The manuscript must describe a technically sound piece of scientific research with data that supports the conclusions. Experiments must have been conducted rigorously, with appropriate controls, replication, and sample sizes. The conclusions must be drawn appropriately based on the data presented.*

*Reviewer #1: Yes*

Thank you very much for your comment.

2. Has the statistical analysis been performed appropriately and rigorously?

Reviewer #1: No

According to the comment, we have analyzed all data statistically using one-way analysis of variance (ANOVA). We have revised the content as follows.

(Page-14)

**Statistical analysis:**

The mean values and standard deviations were calculated from three independent experiments. Statistical analysis was performed using one-way analysis of variance (ANOVA). The values obtained in the experiments were considered to be statistically significant, if P < 0.05.

3. Have the authors made all data underlying the findings in their manuscript fully available?

The [PLOS Data policy](http://www.plosone.org/static/policies.action#sharing) requires authors to make all data underlying the findings described in their manuscript fully available without restriction, with rare exception (please refer to the Data Availability Statement in the manuscript PDF file). The data should be provided as part of the manuscript or its supporting information, or deposited to a public repository. For example, in addition to summary statistics, the data points behind means, medians and variance measures should be available. If there are restrictions on publicly sharing data—e.g. participant privacy or use of data from a third party—those must be specified.

Reviewer #1: Yes

Thank you very much for your comment.

*4. Is the manuscript presented in an intelligible fashion and written in standard English?

PLOS ONE does not copyedit accepted manuscripts, so the language in submitted articles must be clear, correct, and unambiguous. Any typographical or grammatical errors should be corrected at revision, so please note any specific errors here.*

*Reviewer #1: Yes*

Thank you very much for your valuable comment on our manuscript.

*5. Review Comments to the Author

Please use the space provided to explain your answers to the questions above. You may also include additional comments for the author, including concerns about dual publication, research ethics, or publication ethics. (Please upload your review as an attachment if it exceeds 20,000 characters)*

*Reviewer #1: The manuscript is suitable for publication after minor revision:
- Figure 3: in column ligands written 1, 2 and 3 codes for processes but in fig. description the author write a, b and c.*

According to the comment, we have revised the figure 3.

*In single enzyme hydrolysis, please clarify whether the process done in 3 steps or all enzymes together in one step*

According to the comment, we have revised the content as follows.

(Page-9)

**Determination of activity of different enzyme immobilization systems**

For single-enzyme immobilization, each matrix material (Immobilized single-enzymes in sodium alginate) were taken in three different falcon tubes. 10 mL of corn starch solution (1%) was added in the tube containing the α-amylase immobilized matrix and incubated for 30 min at 37 ºC. Then the solution was collected and added in the second tube containing the glucoamylase immobilized matrix and incubated for 30 min at 37 ºC. Then the solution was collected from the second tube and added in the third tube containing the glucose isomerase immobilized matrix and incubated for 30 min at 37 ºC. In case of multi-enzymes immobilization, the immobilized multi-enzyme in sodium alginate beads were taken in a falcon tube and 10 mL of corn starch solution (1%) was added in the tube. For the control experiment, 10 mL of corn starch solution (1%) and 10 mL free enzyme were taken in a falcon tube. All the tubes were incubated for 30 min at 37 ºC. Then 1 mL solution from each tube was taken into new test tubes, and 2 mL of resorcinol solution was added and incubated all test tubes at 80 ºC for 10 min in a water bath. The solution temperature was cooled at room temperature and optical density (OD) of control and sample solutions were measured at 520 nm wavelength using UV-Vis spectrophotometer. The productivity of fructose (mg/mL) was determined from a standard calibration curve. To determine the productivity of fructose by an immobilized enzyme in support matrices and free enzyme, three independent experiments were carried out for each matrix. Average values and standard deviations of productivity of immobilized enzymes in support matrices and free enzymes were calculated.

- All result lack the statistical analysis such as ANOVA

According to the comment, we have analyzed all results statistically using one-way analysis of variance (ANOVA). We also have revised all figures after reanalyzed all results statistically.

- Please improve the resolution of all images

According to the comment, we have revised all figures with higher resolution images.

*6. PLOS authors have the option to publish the peer review history of their article (*[*what does this mean?*](https://journals.plos.org/plosone/s/editorial-and-peer-review-process#loc-peer-review-history)*). If published, this will include your full peer review and any attached files.

If you choose “no”, your identity will remain anonymous but your review may still be made public.*

***Do you want your identity to be public for this peer review?*** *For information about this choice, including consent withdrawal, please see our* [*Privacy Policy*](https://www.plos.org/privacy-policy)*.*

*Reviewer #1: No*

Thank you very much for your comment.
